# Mouse chromosome 2 harbors genetic determinants of resistance to podocyte injury and renal tubulointerstitial fibrosis

**DOI:** 10.1186/s12863-016-0378-1

**Published:** 2016-05-26

**Authors:** Hayato Sasaki, Junpei Kimura, Ken-Ichi Nagasaki, Kiyoma Marusugi, Takashi Agui, Nobuya Sasaki

**Affiliations:** Laboratory of Laboratory Animal Science and Medicine, Faculty of Veterinary Medicine, Kitasato University, Higashi 23-35-1, Towada, 034-8628 Japan; Laboratory of Anatomy, Department of Biomedical Sciences, Graduate School of Veterinary Medicine, Hokkaido University, Kita-18 Nishi-9, Kita-ku Sapporo, 060-0818 Japan; Section of Biological Safety Research, Chitose Laboratory, Japan Food Research Laboratories, Bunkyo 2-3, Chitose, 066-0052 Japan; Laboratory of Laboratory Animal Science and Medicine, Department of Disease Control, Graduate School of Veterinary Medicine, Hokkaido University, Kita-18 Nishi-9, Kita-ku Sapporo, 060-0818 Japan

**Keywords:** Chronic kidney disease, Tensin2, Albuminuria, Renal fibrosis

## Abstract

**Background:**

Tensin2 deficiency results in alterations in podocytes and subsequent glomerular and tubulointerstitial injuries. However, this pathology is critically dependent on genetic background. While the Tensin2-deficient podocytes of resistant murine strains, including C57BL/6J mice, remain almost intact, susceptible murine strains with Tensin2 deficency, including ICGN mice, develop chronic kidney disease following alterations in the podocyte foot processes. In a previous study, genome-wide linkage analysis was utilized to identify the quantitative trait loci associated with the disease phenotypes on mouse chromosome 2. This study investigated the disease phenotypes of chromosome 2 consomic and subcongenic strains.

**Results:**

ICGN consomic mice introgressed with chromosome 2 from the C57BL/6J mouse were generated and found to exhibit milder renal failure than that in ICGN mice. We developed 6 subcongenic strains that carry C57BL/6J-derived chromosomal segments from the consomic strain. One showed significantly milder albuminuria, another showed significantly milder tubulointerstitial injury, and the both showed significantly milder glomerular injury.

**Conclusions:**

These data indicate that mouse chromosome 2 harbors two major genes associated with the severities of nephropathy induced by Tensin2 deficiency. The proximal region on chromosome 2 contributes to the resistance to tubulointerstitial fibrosis. In contrast, the distal region on chromosome 2 contributes to the resistance to podocyte injury. This study would be helpful to discover the biological mechanism underlying the renal injury, and may lead to the identification of therapeutic targets.

**Electronic supplementary material:**

The online version of this article (doi:10.1186/s12863-016-0378-1) contains supplementary material, which is available to authorized users.

## Background

Chronic kidney disease (CKD) is a public-health problem characterized by either kidney damage or a long-term decline in kidney function regardless of disease type or cause, and is becoming increasingly common in both developed and developing countries across the globe [1]. Patients with CKD are at high risk for developing end-stage renal disease (ESRD), requiring costly renal replacement therapy or renal transplantation [1]. In addition, CKD is strongly linked to important diseases such as diabetes, hypertension and cardiovascular disease [2]. Faced with these outcomes, clinical research efforts over the last few decades have focused on preventing or delaying the progression of CKD. Unfortunately, no fundamental therapy is available at present [3].

Taking into account both renal structure and its role in the continuous filtration of blood flow, we can easily imagine that renal glomerular damage due to abnormal blood pressure or components is a potential risk factor for CKD progression. Proteinuria is intimately involved in the dysfunction of glomerular visceral epithelial cells (podocytes) or their intercellular junctions (slit diaphragms), and glomerulosclerosis sequentially starts with a decreased podocyte count (podocytopenia). Glomerulosclerosis is one of the most common histopathological findings of CKD, and mutations in a number of podocyte-specific genes responsible for glomerulosclerosis have been identified in humans [4]. Therefore, podocyte injury is a common determining factor for progression toward many types of kidney disease that result in CKD [5]. Nevertheless, the molecular mechanisms underlying CKD progression remain unclear.

The onset, progression and severity of CKD can be strongly influenced by genetic background. For example, ESRD incidence rates are higher in blacks, Asians and Hispanics in comparison with whites [6]. These racial disparities indicate the presence of modifier genes that prevent or accelerate the progression of CKD. Elucidating the reasons for these differences among diverse genetic backgrounds could help in unraveling the mechanisms for the progression of CKD as well as in developing novel therapies for it, regardless of predisposing factors.

This study approaches CKD with the abovementioned aim in mind, using ICGN mice, a spontaneous CKD mouse model. The null mutation in the tensin2 gene (*Tns2*) is known to be the major causative factor for renal failure in ICGN mice [7], although the function of Tns2 in the kidney and the mechanism by which Tns2 deficiency leads to renal failure remain unknown. Tns2 is a multidomain protein that possesses C1, PTPase, SH2 and PTB domains, and is considered to mediate integrin-associated signaling cascades and regulate Akt signaling [8–11]. Despite its ubiquitous expression and predicted function, Tns2 deficiency results only in alterations in podocytes and subsequent glomerular and tubulointerstitial injuries [7, 12–14]. In addition, the severity of the renal failure caused by Tns2 deficiency is strongly influenced by murine genetic background [11, 14–16]. This genetic background-dependent diversity indicates the presence of modifier genes that prevent renal failure induced by Tns2 deficiency. Recently, we performed a quantitative trait loci (QTL) analysis using backcross progenies from susceptible ICGN and resistant C57BL/6J (B6) strain mice, and identified significant loci for CKD progression on chromosome (Chr) 2 in Tns2-deficient mice [17]. In the present study, to verify the existence of the CKD-resistant loci on Chr 2 from the B6 mouse, we produced ICGN consomic mice homozygously introgressed with Chr 2 from the B6 mouse for the evaluation of CKD progression.

## Methods

### Animals

B6 female mice (Charles River Laboratories Japan, Tokyo, Japan) were backcrossed to ICGN male mice, and consomic ICGN-Chr 2^B6^ mice were generated by repeated marker-assisted backcrossing. The 115 genetic markers used for the Chr substitution between ICGN and B6 mice are shown in Additional file 1: Table S1. The congenic ICGN.B6-*Tns2*^*WT*^ (ICGN-*Tns2*^*WT*^) mice were generated by backcrossing the wild-type *Tns2* from B6 mice into an ICGN background for 10 generations. Genotyping of the *Tns2*^*nph*^ allele (accession MGI:2447990) on Chr 15 was performed as previously described [17]. In order to determine the effect of each QTL on Chr 2, backcrossing of ICGN-Chr 2^B6^ mice to ICGN mice was repeated several times, with six subcongenic strains eventually produced by a marker-assisted speed congenic strategy: ICGN.B6-(*D2Mit1-D2Mit64*) (referred to as ICB2-1), ICGN.B6-(*D2Mit64-D2Mit369*) (ICB2-2), ICGN.B6-*(rs49812762-D2Mit328*) (ICB2-3), ICGN.B6-(*D2Mit378-D2Mit328*) (ICB2-4), ICGN.B6-(*D2Mit219-D2Mit102*) (ICB2-5) and ICGN.B6-(*D2Mit102-D2Mit451*) (ICB2-6) strains. All strains were born in accordance with Mendelian inheritance at an equal sex ratio. The additional genetic markers for fine mapping of introgressed genomic intervals of the six subcongenic strains are shown in Fig. 4 and Additional file 1: Table S2. The map positions of the microsatellite markers were based on information from the Mouse Genome Informatics of the Jackson Laboratory (MGI; http://www.informatics.jax.org/, MGI_4.41). The animal facility was air-conditioned at 22 ± 2 °C, maintained at 40–60 % relative humidity, and mice were maintained under a 12 h light-dark cycle. A standard laboratory diet, Labo MR Standard (Nosan, Kanagawa, Japan), and tap water were provided *ad libitum*. The animals’ microbiological status was monitored periodically according to Japanese Association of Laboratory Animal Facilities of Public and Private Universities guidelines. A humane end point was applied when the mice with severe anemia became moribund. Male virgin mice were used for all analyses.

### Measurements of blood

Blood samples from 16-week-old mice were collected from the inferior vena cava under 4 % isoflurane anesthesia. Hemoglobin concentration (Hb), hematocrit (Ht), red blood cell count (RBC) and blood urea nitrogen (BUN) were measured using an automated counter (KX-21NV; Sysmex Corporation, Kobe, Japan) and an automated biochemical analyzer (Dri-Chem 7000; Fuji Photo Film, Tokyo, Japan) as described previously [15].

### Measurement of urinary albumin excretion

Urine samples were collected by gentle manual compression of the abdomen at 4, 8, 16 and 28 weeks of age. A 10-μL aliquot (containing 2 % sodium dodecyl sulfate (SDS), 5 % β-mercaptoethanol, 10 % glycerol, 60 mM Tris-HCl (pH 6.8), bromophenol blue and 1 μL of urine) was heated for 5 min at 95 °C and applied to 10 % SDS-polyacrylamide gel electrophoresis. As a positive control, 5 μg of bovine serum albumin was loaded simultaneously. The gel was fixed and stained with Coomassie brilliant blue (CBB; Wako, Osaka, Japan) according to manufacturer’s instructions, and scanned by a standard commercial scanner. CBB-stained urinary albumin was quantified by the gel analysis program of ImageJ (http://imagej.net/).

### Histological damage scores

Formalin-fixed and paraffin-embedded kidney blocks were sectioned at a thickness of 2 μm, and stained with periodic acid-Schiff (PAS) solution. The severity of renal damage was quantitated using two types of histopathological score (a glomerular index and tubular index) as previously described [14, 17]. The glomerular index, based on the progression of glomerulosclerosis, was calculated as an average of the following ratings for 20 randomly selected glomeruli: 0, no abnormality; 1, mild expansion of the mesangial matrix; 2, partial thickening of glomerular basement membrane (GBM); 3, vascular stenoses (partial expansion of the mesangial matrix); 4, entire expansion of the mesangial matrix; and 5, abnormal dilation of capillary lumen or retraction and collapse of the glomerular tuft [14]. To provide a more precise definition of the progression of tubulointerstitial fibrosis, we modified the previously described tubular index. The modified tubular index is the sum of the following observations: 1, mild tubular dilations or 2, focal tubular dilations or 3, massive tubular dilations; 1, huge dilation (>200 μm) of the tubules with urinary casts; 1, interstitial cell expansion or 2, severe interstitial cell expansion (Additional file 1: Figure S1). Zero points signify the absence of any abnormality. The mice were analyzed at 16 weeks of age as ICGN mice show severe albuminuria and anemia, which are the end stages of a variety of pathological conditions.

### Evaluation of tubulointerstitial fibrosis

Formalin-fixed and paraffin-embedded kidney blocks were sectioned at a thickness of 3 μm, and processed for Masson’s trichrome (MT) staining. Five random renal cortex fields for each sample were chosen, and the aniline blue (AB)-stained area, representing interstitial collagen spread, was measured using ImageJ software.

### Immunohistochemical analysis

Kidneys from 16-week-old mice were fixed with 10 % buffered formalin at 4 °C overnight. The formalin-fixed paraffin sections (2-μm thick) were subjected to normal histological processes and antigen retrieval with citrate buffer (pH 6.0). After treatment with 3 % H_2_O_2_ and blocking with normal donkey serum for 1 h, sections were incubated with goat anti-mouse IL-1F6 antibody (1:400 diluted, R&D Systems, Minneapolis, MN, USA) at 4 °C overnight. Sections were then incubated with biotin-conjugated donkey anti-goat IgG secondary antibody (Santa Cruz Biotechnology, Dallas, TX, USA) for 30 min at room temperature, and treated with horseradish peroxidase-conjugated streptavidin complex (Histofine; Nichirei Biosciences, Tokyo, Japan) for 3,3-diaminobenzidine (DAB) staining. Five random renal cortex fields for each sample were chosen, and the signal intensity of DAB was measured using ImageJ software.

### Ultrastructural analysis

Kidneys from 16-week-old mice were cut into small pieces (1 mm^3^) and prefixed in 2.5 % buffered glutaraldehyde for 4 h, and then fixed with 1 % buffered osmium tetroxide for 2 h. Fixed tissues were dehydrated by graded alcohol, and embedded in epoxy resin (Quetol 812 Mixture; Nisshin EM, Tokyo, Japan). Epoxy resin-embedded specimens were sectioned at a thickness of 70 nm, stained with uranyl acetate and lead citrate, and observed under a JEOL transmission electron microscope (JEM-1210; JEOL, Tokyo, Japan).

### Statistics

Data are expressed as means ± standard deviation. Statistical analyses were performed with Student’s *t*-test, Mann-Whitney U test, Dunnett’s multiple comparison test and Dunn’s multiple comparison test. *P* <0.05 was considered significant.

## Results

### Verification of CKD-resistant loci on chromosome 2

**Fig. 1:**
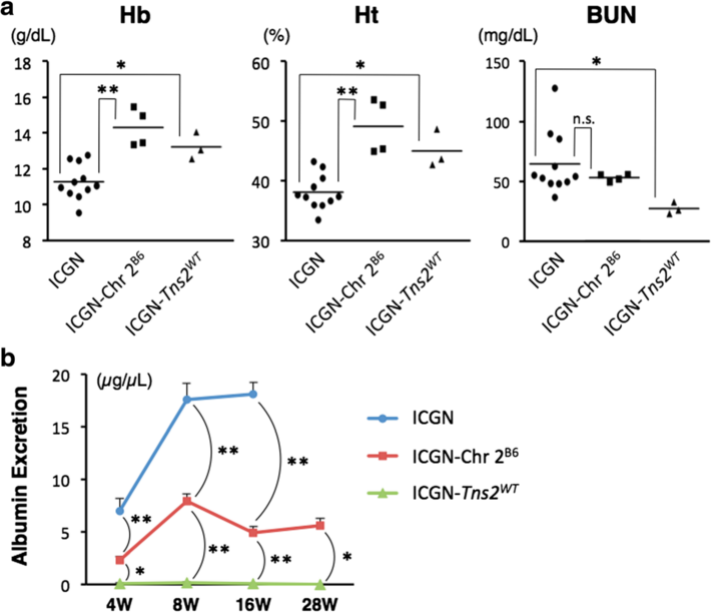
Measurements of blood and urine. **a** Hematologic parameters at 16 weeks of age. The levels of Hb and Ht in ICGN-Chr 2^B6^ mice and ICGN-*Tns2* ^*WT*^ mice were normal. With regard to BUN, no significant difference was observed between ICGN and ICGN-Chr 2^B6^ mice, with some ICGN mice but no ICGN-Chr 2^B6^ mice showing abnormal BUN levels. **b** Urinary albumin excretion. Albuminuria was already observed at 4 weeks after birth in ICGN and ICGN-Chr 2^B6^ mice. Asterisks indicate *P*-values for Dunnett’s multiple comparison test, ** <0.01 and * <0.05 vs. ICGN mice (**a**), or ICGN-Chr 2^B6^ mice at the same time point (**b**) n.s., not significant. *Error bars* represent standard error. Eleven ICGN mice, four ICGN-Chr 2^B6^ mice and three ICGN-*Tns2* ^*WT*^ mice were analyzed

For verification of the existence of the CKD-resistant loci on Chr 2 from the B6 mice, which are linked to all tested parameters for CKD, we produced ICGN consomic mice homozygously introgressed with Chr 2 (*D2Mit1-D2Mit229*, 2.23–88.99 cM) from the B6 mouse (ICGN-Chr 2^B6^) using marker-assisted backcrossing. For phenotype analyses of the ICGN-Chr 2^B6^ mice, we produced a congenic mouse possessing an ICGN genetic background by replacing *Tns2*^*nph*^ with wild-type Tns2 (ICGN-*Tns2*^*WT*^) as a control strain not affected by nephropathy. The ICGN, ICGN-Chr 2^B6^ and ICGN-*Tns2*^*WT*^ phenotypes were then analyzed. ICGN mice showed low Hb (11.3 ± 0.9 mg/dL) and low Ht (38.2 ± 2.8 %) (Fig. 1a). On the other hand, the erythrocytic parameters of the ICGN-Chr 2^B6^ mice as well as those of the ICGN-*Tns2*^*WT*^ were at normal levels (Hb, 14.3 ± 1.0 mg/dL; Ht, 49.1 ± 4.6, Fig. 1a). There was no significant difference in BUN between the ICGN and ICGN-Chr 2^B6^ mice, with no ICGN-Chr 2^B6^ mice and only a few ICGN mice showing an abnormal BUN level (Fig. 1a). Both ICGN and ICGN-Chr 2^B6^ mice developed albuminuria by 8 weeks of age, but the urinary albumin excretion in ICGN-Chr 2^B6^ mice was always lower than that in ICGN mice (4.8 ± 1.1 μg/μL vs. 18.1 ± 2.7 μg/μL at 16 weeks old, Fig. 1b).

**Fig. 2:**
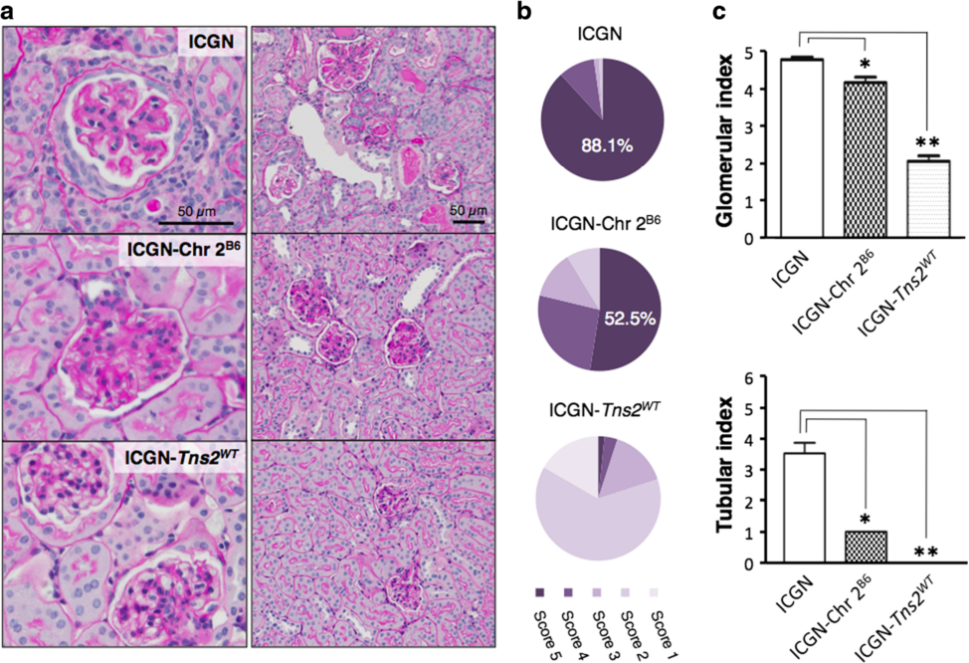
Histological analyses of PAS-stained renal sections from 16-week-old mice. **a** Representative glomerular and cortical sections. ICGN: entire expansion of the mesangial matrix and vascular dilations (score 5), massive tubular dilations with urinary casts and cell infiltrations. ICGN-Chr 2^B6^: partial expansion of the mesangial matrix and vascular stenoses (score 3). ICGN-*Tns2* ^*WT*^: nearly normal (upper, score 0), partial expansion of the mesangial matrix and vascular wall thickening (lower, score 2). **b** The mean ratio of the glomerular injury scores each sample. **c** Glomerular injury index and tubular injury index. Asterisks indicate *P*-values for Dunn’s multiple comparison test, ** <0.01 and * <0.05 vs. ICGN mice. *Error bars* represent standard error. Eleven ICGN mice, four ICGN-Chr 2^B6^ mice and three ICGN-*Tns2* ^*WT*^ mice were analyzed

**Fig. 3:**
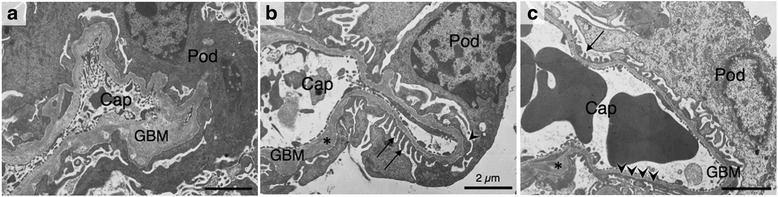
Ultrastructure of the glomeruli at 16 weeks of age. In ICGN mice (**a**), high-level effacement of the podocyte foot processes, high-level thickening of the GBM and swelling of the podocytes (Pod) were observed. In ICGN-Chr 2^B6^ mice (**b**), normal GBM and foot processes (*arrow*) as well as thickening of the GBM (asterisk) and effacement of the foot processes (*arrowhead*) were observed with a much milder phenotype than that of ICGN mice. In ICGN-*Tns2* ^*WT*^ mice (**c**), the glomeruli were nearly normal, but some part of the foot processes and the GBM was abnormal. Cap, capillary

Histopathologic analysis of the kidney sections revealed that ICGN-Chr 2^B6^ mice showed a milder phenotype than did ICGN mice (Figs. 2 and 3). Injury severity scores varied widely in the glomeruli of ICGN-Chr 2^B6^ mice, whereas almost all of the glomeruli of ICGN mice showed entire expansion of the mesangial matrix or collapse of the glomerular tufts (score 5, also see Fig. 2). Massive dilations of the proximal tubules with urinary casts, interstitial cell expansion and accumulation of interstitial collagen were observed in ICGN mice, but not in ICGN-Chr 2^B6^ mice up to 16 weeks old (Fig. 2 and Additional file 1: Figure S2). These tubulointerstitial lesions were mainly found in renal cortex. In addition, the ratio of the IL-1 F6-positive area, a marker of tubulointerstitial lesions [18], in the renal cortex parenchymal tissue of congenic mice was less than that for the ICGN mice (1.53 ± 0.22 % vs. 0.30 ± 0.17 %, Additional file 1: Figure S3). Indeed, In ICGN-Chr 2^B6^ mice, marked injuries as found in 16-week old ICGN mice were detected up to 28 weeks old, indicating that ICGN-Chr 2^B6^ mice developed tubulointerstitial fibrosis slowly compared with ICGN mice (Fig. 2 and Additional file 1: Figure S2, S4). Electron microscope observation also showed that the ultrastructural glomerular alterations in the ICGN-Chr 2^B6^ mice were milder than those in the ICGN mice (Fig. 3).

ICGN-*Tns2*^*WT*^ mice did not develop albuminuria up to 28 weeks old, but unexpectedly, displayed slight foot process effacement and GBM thickening in a few glomeruli (Figs. 1b and 3c). In addition, ICGN-*Tns2*^*WT*^ mice showed noticeable focal tubular dilation without collagenous thickening (score 1, Additional file 1: Figure S4).

### Genetic mapping for the resistance to the nephropathy

**Fig. 4:**
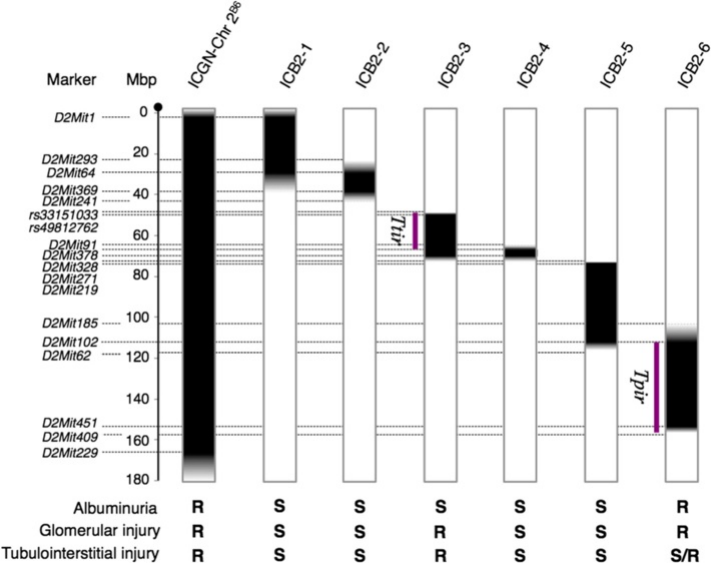
Chromosome 2 genotypes and phenotypic summary of the congenic strains. The white and black bars indicate the genomic region from ICGN and B6 mice, respectively. The shaded bar shows an unclear genomic region in which recombination occurred. If a phenotypic severity was significantly alleviated by genetic recombination compared to that of ICGN mice (see Fig. 5 and Additional file 1: Figure S2), it is defined as resistant (R). If not, it is defined as susceptible (S). *Tns2* ^*nph*^-induced tubulointerstitial fibrosis related locus, *Ttir*, is located in *rs33151033-D2Mit378* (49.5–68.8 Mb). *Tns2* ^*nph*^-induced podocyte injury related locus, *Tpir*, is located in *D2Mit102-D2Mit409* (114.1–158.5 Mb)

**Fig. 5:**
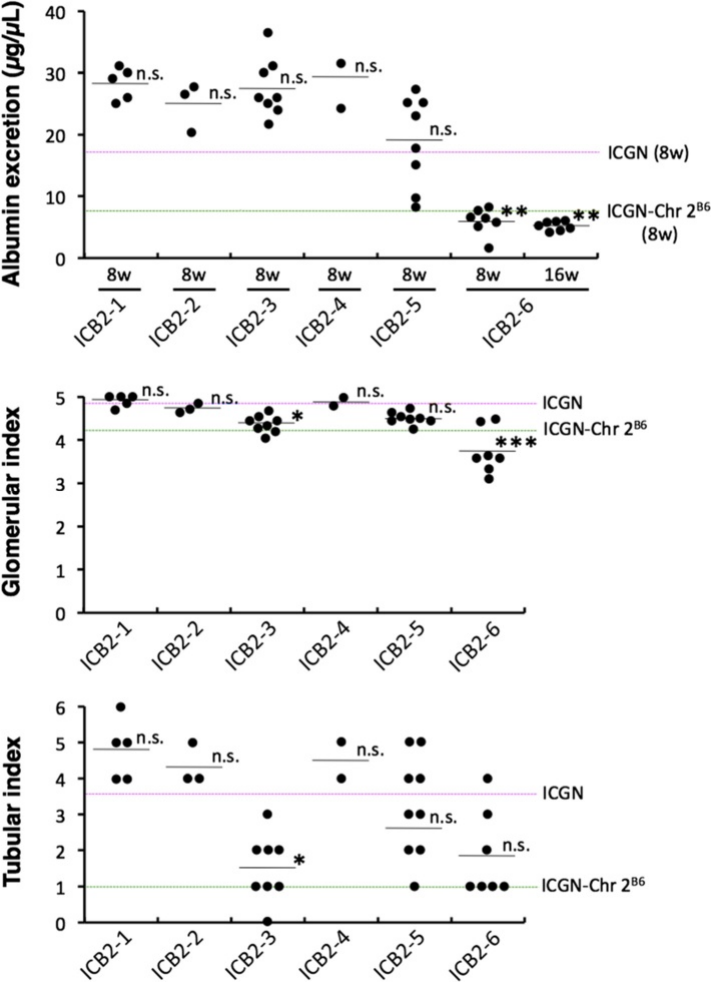
Urinary albumin excretion, glomerular index and tubular index of the congenic strains. ICGN-Chr 2^B6^ and ICGN data are identical to Figs. 1b and 2. Statistical analysis was performed for ICGN and all subcongenic strains. Dunn’s and Dunnett’s multiple comparison test were used for statistical analyses of glomerular and tubular injury indexes, and urinary albumin excretion, respectively. Asterisks indicate *P*-values, *** <0.001, ** <0.01 and * <0.05 vs ICGN mice. n.s., no significant differences compared with ICGN mice. Error bars represent standard error. Five ICB2-1 mice, three ICB2-2 mice and eight ICB2-3 mice, two ICB2-4 mice, eight ICB2-5 mice and seven ICB2-6 mice were analyzed

In order to map the CKD-resistant loci on Chr 2 from the B6 mice, we generated six subcongenic strains that carried a genomic segment of Chr 2 of B6 mice from ICGN-Chr 2^B6^. The genomic regions from the B6 mice and their phenotypic summary are shown in Fig. 4. Among these subcongenic strains, only ICB2-6 mice and ICGN-Chr 2^B6^ mice exhibited significantly lower urinary albumin excretion than ICGN mice at 8 weeks of age (Fig. 5). Even at 16 weeks old, urinary albumin excretion in ICB2-6 mice remained at a low level similar to that in ICGN-Chr 2^B6^ mice (Figs. 1b and 5). On the other hand, only ICB2-3 mice among these subcongenic strains exhibited a significantly lower tubular index than that in ICGN mice (Fig. 5). Although there were no significant differences in tubular index between ICGN and the subcongenic strains except ICB2-3, ICB2-6 mice also showed milder tubulointerstitial lesions than did ICGN mice (tubular index 1.8 ± 1.2 vs. 3.5 ± 1.1, Fig. 5). With regard to the glomerular index and renal collagenous thickening as assessed by MT staining, both ICB2-3 and ICB2-6 mice exhibited significantly milder phenotypes than did ICGN mice (Fig. 5 and Additional file 1: Figure S2).

## Discussion

We previously reported the mapping of genetic loci responsible for resistance to congenital nephropathy caused by a Tns2 deficiency in a B6 genetic background. Analysis of backcrossing between resistant B6 mice and susceptible ICGN mice has identified significant QTLs on Chrs 2 and 13 [17]. Among the tested traits, tubulointerstitial injury (tubular index) and anemia (Hb and Ht) were strongly associated with the same locus on Chr 2, possibly because, in patients with CKD, anemia develops due to decreased renal erythropoietin synthesis resulting from tubulointerstitial damage [16]. In fact, the ICGN consomic mice carrying B6-derived Chr 2 (ICGN- Chr 2^B6^) exhibited a milder phenotype than did the ICGN mice, indicating the existence of a CKD-related locus on Chr 2 (Figs. 1, 2 and 3 and Additional file 1: Figure S2, S3).

Next, we produced six subcongenic strains from ICGN-Chr 2^B6^ mice for genetic mapping of the CKD-related locus on Chr 2. Interestingly, phenotypic analysis of kidney disease in the subcongenic mice revealed that two strains have a resistant phenotype (Fig. 4). ICB2-6 mice exhibited lower urinary albumin excretion, at a level similar to that of ICGN-Chr 2^B6^ mice, than did ICGN mice (Fig. 5). ICB2-3 mice as well as ICGN mice developed massive albuminuria, but ICB2-3 mice exhibited milder tubulointerstitial fibrosis than did ICGN mice (Fig. 5 and Additional file 1: Figure S2). These results suggest that two genetic loci on mouse Chr 2 independently confer resistance to CKD, and contribute to the ICGN-Chr 2^B6^ mouse phenotypes; i.e., ICB2-6 and ICB2-3 mice show resistance to albuminuria (i.e., podocyte injury) and tubulointerstitial fibrosis, respectively. The milder glomerular phenotype of ICB2-6 mice is considered to be due to their resistance to podocyte injury and subsequent proteinuria and glomerulosclerosis (Fig. 5) [19]. Tubulointerstitial fibrosis, the final common pathway of CKD leading to ESRD, is formed via the epithelial-mesenchymal transition of tubular epithelial cells in a complex cellular and molecular milieu caused by glomerular proteinuria [20–23]. Tubulointerstitial fibrosis thereby leads to secondary glomerular injury [24]. These renal pathologies are thought explain why ICB2-6 mice and ICB2-3 mice exhibited milder histological phenotypes in terms of tubular and glomerular index, respectively (Fig. 5 and Additional file 1: Figure S2). In contrast, ICB2-4 and ICB2-5 mice, which possess a genomic interval from B6 mice that overlaps with the genome of both ICB2-3 and ICB2-6 mice, did not show a resistant phenotype.

From the results of our congenic line analysis, we mapped the genomic intervals associated with resistance to podocyte injury and tubulointerstitial fibrosis in Tns2-deficiet mice, and designated the former locus *Tpir*, *Tns2*^*nph*^-induced podocyte injury resistance, and the latter locus *Ttir*, *Tns2*^*nph*^-induced tubulointerstitial fibrosis resistance (Fig. 4). The region conferring tubulointerstitial fibrosis resistance, *Ttir*, covers the LOD peak positions of QTLs for resistance to tubulointerstitial injury and renal anemia in Tns2-deficient mice (31 and 36 cM) [17]. As with many nephropathy model rodents, tubulointerstitial fibrosis in ICGN mice is considered to be a secondary lesion following early podocyte injury [13, 14], thus the resistant effect on tubulointerstitial fibrosis should potentially work irrespective of cause. The gene responsible for *Ttir* is, therefore, speculated to help universally block CKD progression toward ESRD. Similar approaches have been attempted in human CKD, but the efficacies against renal fibrosis proven in experimental animal models have not been successfully demonstrated in human trials [25]. The likely reason for this is that renal fibrosis in experimental animal models, such as the unilateral ureteral obstruction (UUO) model and nephrectomy model, only appears similar to that in human CKD while being essentially different from it. CKD in ICGN mice progresses gradually from continuous glomerular injury to tubulointerstitial fibrosis like human CKD, while experimental animal models display acute tubulointerstitial fibrosis, which is rather close to patients with acute kidney injury. Actually, B6 mouse was found to be resistant to renal fibrosis in bovine serum albumin (BSA)-injected proteinuria model, but be susceptible to renal fibrosis in UUO model [26, 27]. From this perspective as well, the gene responsible for *Ttir* may shed light on novel molecular mechanisms associated with renal fibrosis, although *Ttir* does not overlap with previously reported genetic loci associated with renal fibrosis or ESRD.

While ICGN-Chr 2^B6^ mice displayed lower urinary albumin excretion and milder ultrastructural alterations in podocytes than did ICGN mice (Figs. 1b and 3), the Tns2-deficient mice possessing the genetic background of the B6 or 129^+*Ter*^/SvJcl (129 T) mice do not even develop albuminuria [11, 15, 16]. Moreover, although B6 congenic mice carrying the *Tns2*^*nph*^ mutation exhibit slight GBM thickening and mild mesangial expansion, the morphology of the podocytes remains almost intact up to 40 weeks of age [11]. That is, in terms of the primordial prevention of podocyte injury induced by *Tns2*^*nph*^, the effects of the podocyte injury-resistant region, *Tpir*, on Chr 2 from the B6 mouse were insufficient in comparison with those of the B6 genetic background itself. This suggests that other loci outside of Chr 2 confer immediate resistance to Tns2 deficiency.

While both B6 and 129T mice are resistant to Tns2 deficiency, B6 mice also exhibit lower albuminuria in hypertension or diabetes model than do 129 strains [28, 29]. Indeed, B6 mice appear to be resistant to various podocyte injuries caused not only by Tns2 deficiency but also by hypertension, diabetes, angiotensin II infusion, HIV-associated nephropathy (HIVAN), albumin overload, renal ablation or congenital nephron reduction [28–36]. These findings lead to the hypothesis that the B6 genetic background possesses a “pan-resistance” to various podocyte injuries that works through the protection of the podocytes themselves. With regard to HIVAN, Papeta et al. reported that the transcript level of *Nphs2*, which encodes podocin, was linked to strain-dependent resistance, and B6 and 129/SvEv mice displayed 3-fold higher expression of *Nphs2* than did FVB and D2 mice [33]. The abundance of podocin, a scaffolding molecule for the slit diaphragm, in B6 podocytes can be interpreted to mean that B6 mice have “robust” podocyte foot processes that are resistant to injury. This concept may be applicable to *Tpir*. It is interesting that *Tpir* overlaps with the QTLs for age-associated albuminuria in crosses between B6 and D2 or A/J mice (*Albq5* and *Albq1*), and the common genomic interval (129–158 Mb) includes the LOD peak positions of these QTLs (Additional file 1: Figure S5) [37, 38]. Normal D2 and A/J mice, which don’t carry a marked risk factor for renal failure, develop mild albuminuria with increasing age [37, 38], but this age-associated phenotype appears to be common in human CKD [39, 40]. As for age-associated albuminuria, the age-related decrease in the uptake of glomerular filtrate albumin by renal proximal tubule as well as glomerular damage can contribute to albumin leak [41]. Age-related change of the expression of megalin and cubilin, major receptors responsible for the reabsorption of albumin, was found in old rats [41]. However, neither genes are located in major QTLs, *Albq5*, *Tpir* and *Ttir*. It is also notable that the distal region of *Tpir* (154–157 Mb) includes the syntenic region of human Chr 20q11.22, which is linked to the estimated glomerular filtration ratio (eGFR) [42], and the proximal region of *Tpir* includes *Ino80* (119 Mb), which has been identified with single nucleotides polymorphisms associated with eGFR in European ancestry samples [43], however, *Tpir* is still very broad. Therefore, further studies are needed to narrow the genomic interval of *Tpir*, and investigate whether *Tpir* contributes to a “pan-resistance” to various podocyte injuries in B6 genetic background.

On the other hand, we unexpectedly discovered that ICGN control mice possessing wild-type *Tns2* (ICGN-*Tns2*^*WT*^) displayed slight alterations in the podocyte foot processes, GBM and focal tubular dilations without interstitial fibrosis at 16–28 weeks of age (Fig. 3c and Additional file 1: Figure S4). These findings indicate that ICGN mice carry a few minor causative factors for nephropathy in addition to *Tns2*^*nph*^.

## Conclusions

Our congenic analysis using Tns2-deficient mice demonstrated the existence of QTLs for CKD resistance mapped to Chr 2 in our previous study, and identified that the Chr 2 QTLs were comprised of two genetic regions, *Tpir* and *Ttir*, associated with resistance to podocyte injury and tubulointerstitial fibrosis, respectively. As the resistance to these conditions appears to have a protective effect on renal injuries irrespective of the mechanism by which Tns2-deficiency leads to renal failure, the genes responsible for this resistance can help protect the kidney from various renal injuries irrespective of cause and aid in the development of universally effective therapies for CKD. Further studies are required to understand the exact mechanism of the resistance/susceptibility to renal disease in our strains. Nevertheless, the present study provides a first step in the identification of novel causative gene(s) that might lead to the development of treatment strategies for CKD.

## Abbreviations

AB, aniline blue; BUN, blood urea nitrogen; Chr, chromosome; CKD, chronic kidney disease; cM, centimorgan; DAB, 3,3-diaminobenzidine; eGFR, estimated glomerular filtration ratio; ESRD, end-stage renal disease; GBM, glomerular basement membrane; Hb, hemoglobin concentration; HIVAN, HIV-associated nephropathy; Ht, hematocrit; Mb, megabase; MT, Masson’s trichrome; QTL, quantitative trait loci; RBC, red blood cell count; UUO, unilateral ureteral obstruction

## Additional file

Additional file 1:
**Table S1.** Genotyping markers for consomic analysis. **Table S2.** Genotyping markers for subcongenic strains. **Figure S1.** Representative examples of modified tubular score. **Figure S2.** Quantitative analysis of tubulointerstitial fibrosis. **Figure S3.** Quantitative analysis of tubulointerstitial damage. **Figure S4.** Histological analyses of PAS-stained and MT-stained renal sections from 28-week-old mice. **Figure S5.** QTL map for urinary albumin excretion on mouse Chr 2 and the genomic interval of *Tpir*. (PDF 26631 kb)
