# Supplementary material for: Mouse chromosome 2 harbors genetic determinants of resistance to podocyte injury and renal tubulointerstitial fibrosis
Source: BMC Genet. 2016 May 26;17:69. doi: 10.1186/s12863-016-0378-1 (PMC4882790; doi:10.1186/s12863-016-0378-1)
Supplement: Additional file 1: — Table S1. Genotyping markers for consomic analysis. Table S2. Genotyping markers for subcongenic strains. Figure S1. Representative examples of modified tubular score. Figure S2. Quantitative analysis of tubulointerstitial fibrosis. Figure S3. Quantitative analysis of tubulointerstitial damage. Figure S4. Histological analyses of PAS-stained and MT-stained renal sections from 28-week-old mice. Figure S5. QTL map for urinary albumin excretion on mouse Chr 2 and the genomic interval of Tpir. (PDF 26631 kb) [file 12863_2016_378_MOESM1_ESM.pdf]

Additional file 1

Mouse chromosome 2 harbors genetic determinants of resistance to podocyte injury and renal tubulointerstitial fibrosis.

Hayato Sasaki[1], Junpei Kimura[2], Ken-Ichi Nagasaki[3], Kiyoma Marusugi[1], Takashi Agui[4] and Nobuya Sasaki[1]*

1. Laboratory of Laboratory Animal Science and Medicine, Faculty of Veterinary Medicine, Kitasato University, Higashi 23-35-1, Towada 034-8628, Japan.
2. Laboratory of Anatomy, Department of Biomedical Sciences, Graduate School of Veterinary Medicine, Hokkaido University, Kita-18 Nishi-9, Kita-ku, Sapporo 060-0818, Japan.
3. Section of Biological Safety Research, Chitose Laboratory, Japan Food Research Laboratories, Bunkyo 2-3, Chitose 066-0052, Japan.
4. Laboratory of Laboratory Animal Science and Medicine, Department of Disease Control, Graduate School of Veterinary Medicine, Hokkaido University, Kita-18 Nishi-9, Kita-ku, Sapporo 060-0818, Japan.

* Corresponding author Address: (Nobuya Sasaki)
Laboratory of Laboratory Animal Science and Medicine
School of Veterinary Medicine, Kitasato University,
Higashi-23-35-1, Towada 034-8628 Japan
Phone/ Fax: +81-176-24-9496
E-mail: nobsasa@vmas.kitasato-u.ac.jp

Figure S1. Representative examples of modified tubular score.

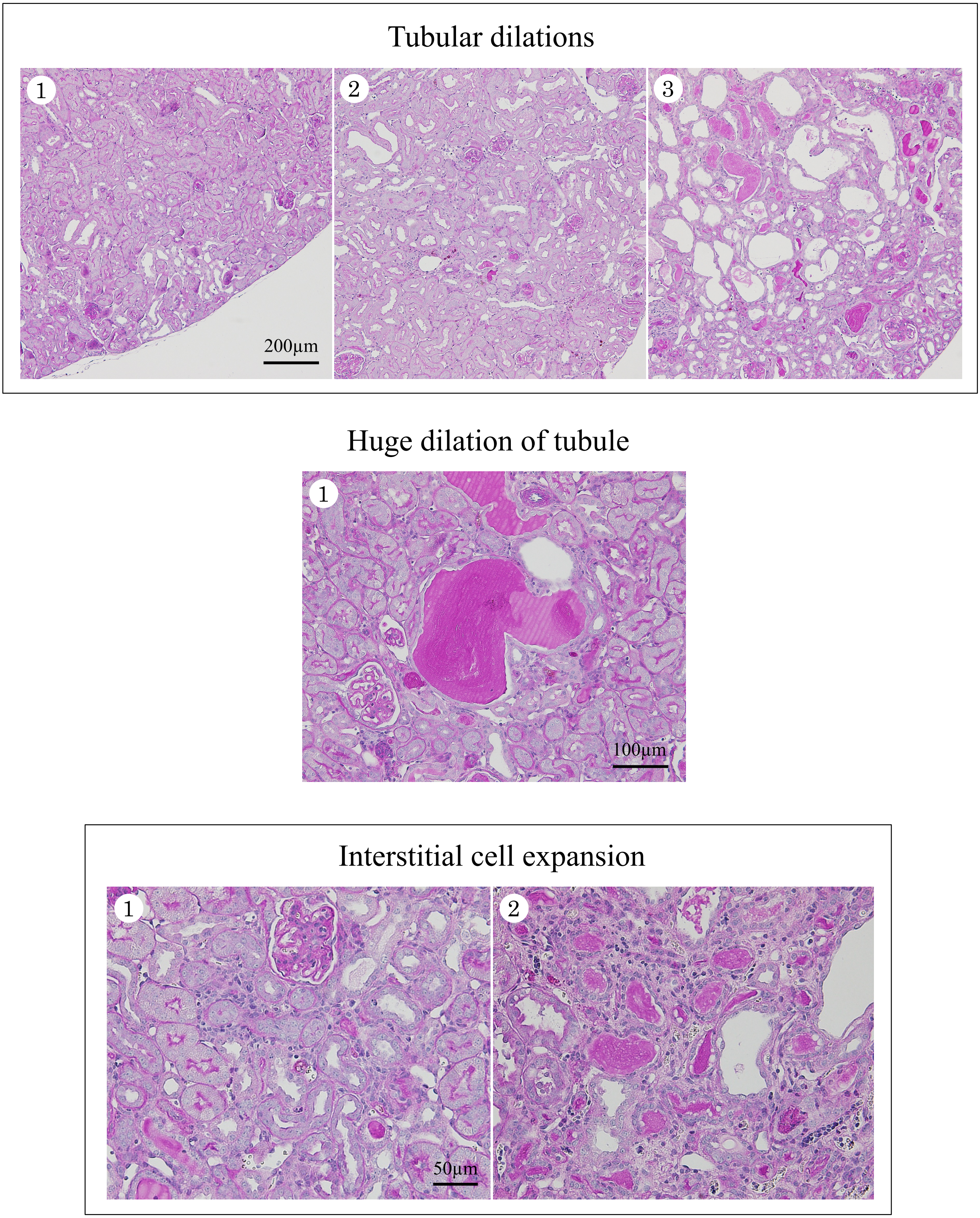

Figure S2. Quantitative analysis of tubulointerstitial fibrosis.

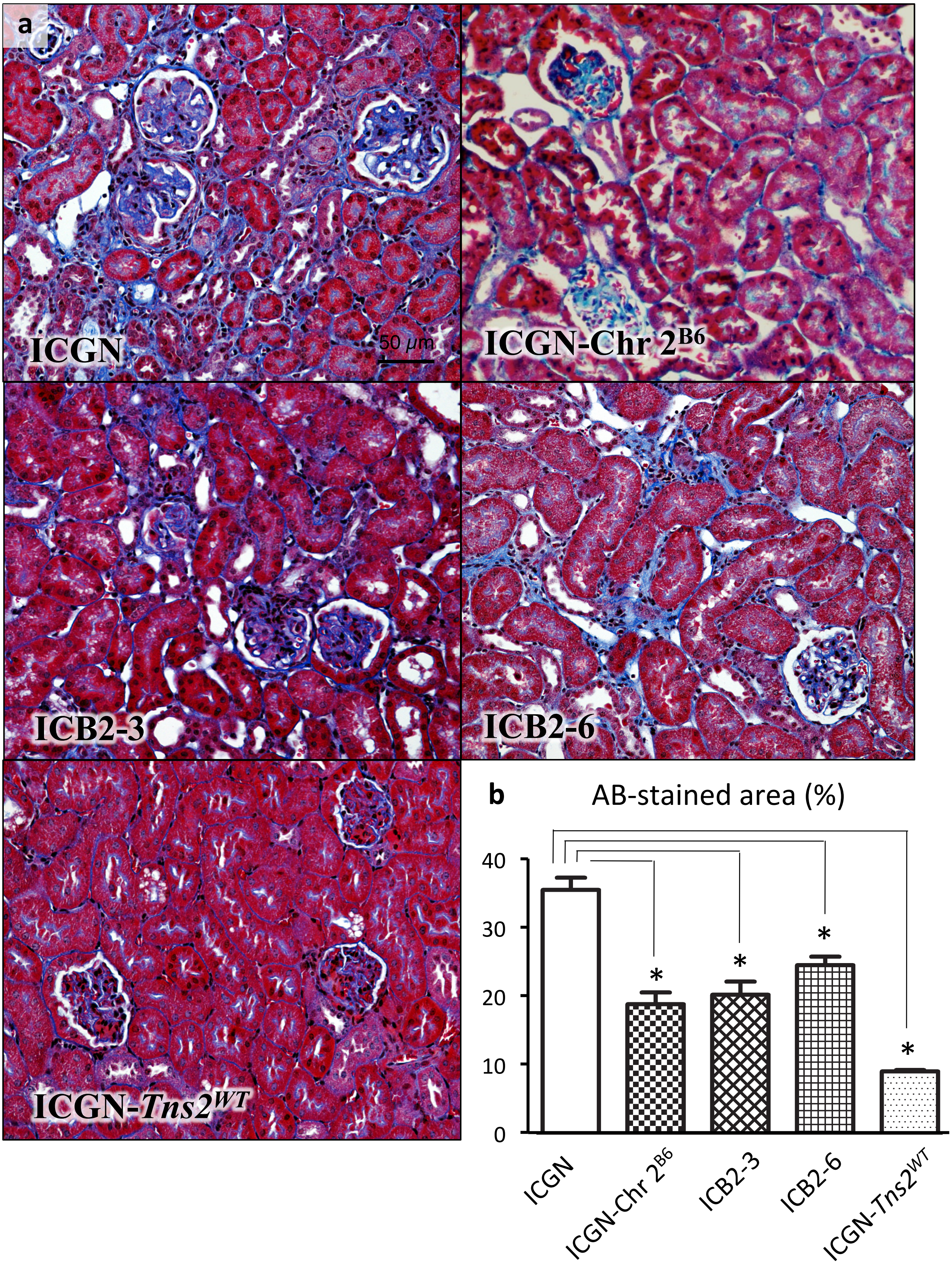

(a) MT-stained renal cortical sections from 16-week-old mice. (b) The mean ratio of the AB-stained area in the renal cortex parenchymal tissue was calculated for each sample. Asterisk indicates P-value vs. ICGN mice for Dunnett's multiple comparison test (P< 0.001). Error bars represent standard error. Eight ICGN mice, four ICGN-Chr 2^{B6} mice, six ICB2-3 mice, five ICB2-6 mice and three ICGN-*Tns2*^{WT} mice were analyzed.

Figure S3. Quantitative analysis of tubulointerstitial damage.

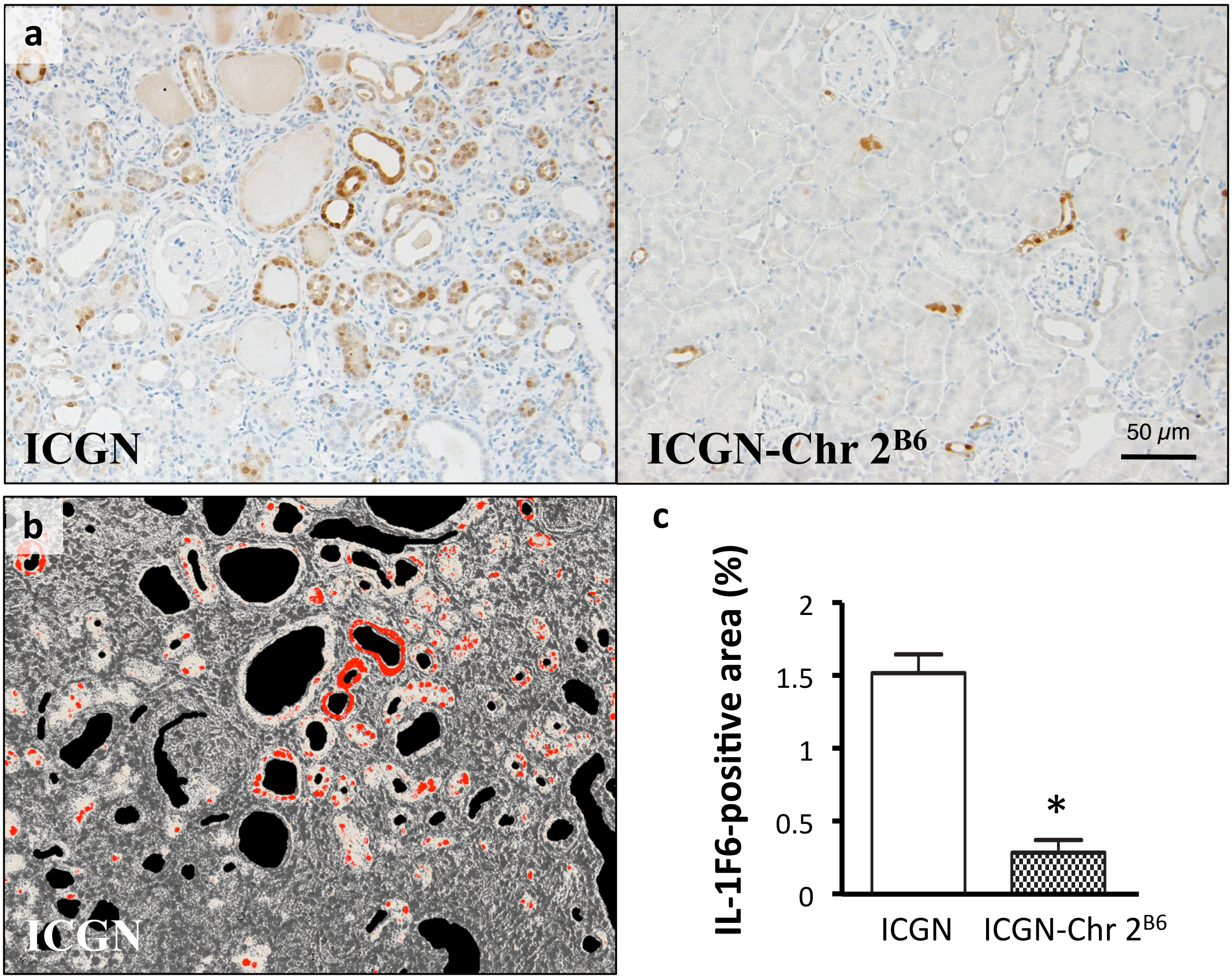

(a) Kidney sections from 16-week-old mice were immunostained with anti-IL-1F6 antibody.
(b) The corresponding quantitative image for the IL-1F6-positive area (red), a marker of tubulointerstitial lesions. (c) The mean ratio of the IL-1F6-positive area in the renal cortex parenchymal tissue was calculated for each sample. The results were consistent with those of the tubular injury index (see Figure 2). Asterisk indicates P-value for Student's t-test ($P < 0.01$). Error bars represent standard error. N = 4.

Figure S4. Histological analyses of PAS-stained and MT-stained renal sections from 28-week-old mice.

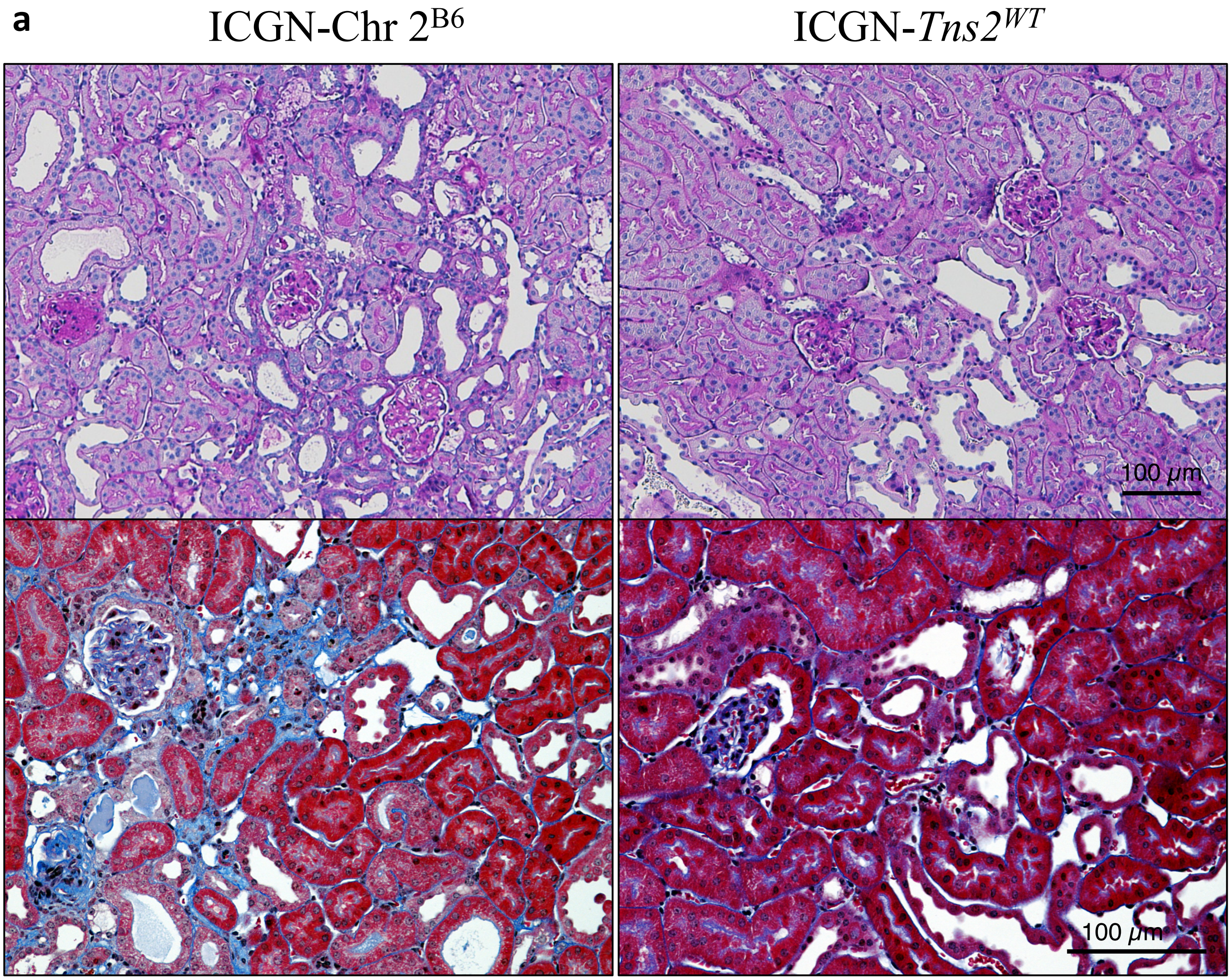

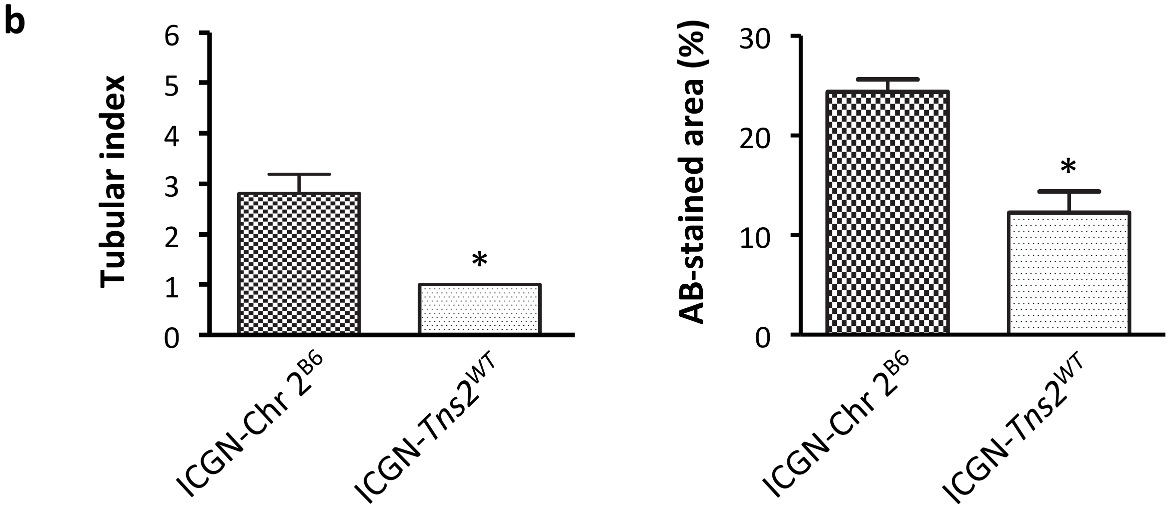

(a) PAS-stained (upper) and MT-stained (lower) renal cortical sections.

(b) Tubular injury index and the mean percentage of the AB-stained area in the renal cortex parenchymal tissue. Mann-Whitney U test and Student's t-test were used for statistical analyses of tubular injury index and AB-stained area, respectively. Asterisk, $P < 0.05$. Error bars represent standard error. Five ICGN-Chr 2^{B6} mice and three ICGN-*Tns2*^{WT} mice were analyzed.

Figure S5. QTL map for urinary albumin excretion on mouse Chr 2 and the genomic interval of *Tpir*.

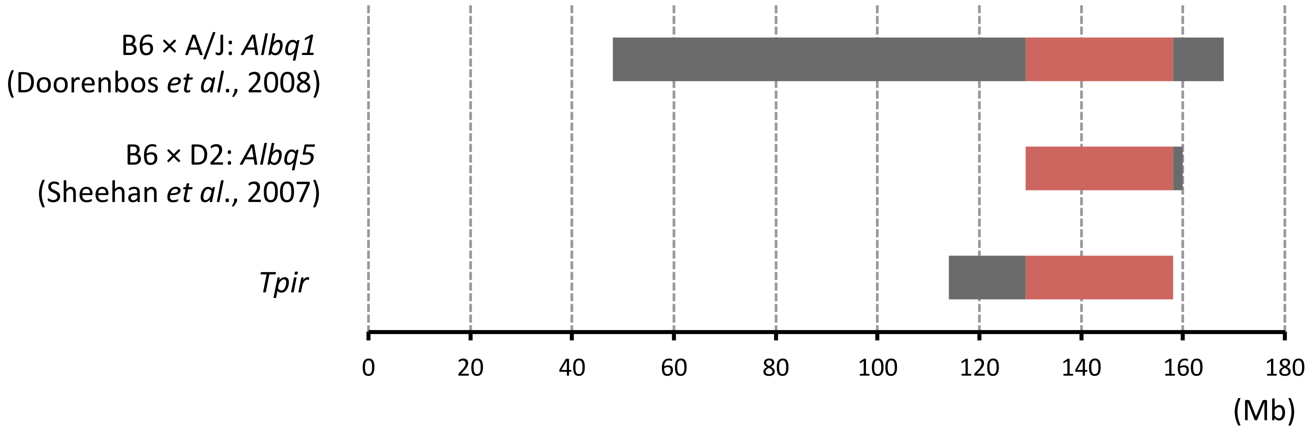

Albq1 (LOD peak 141 Mbp, 95% confidence interval 48-168 Mbp) was identified as QTL for albuminuria in a cross between B6 and A/J mice [37]. *Albq5* (LOD peak 150 Mbp, 95% confidence interval 129-160 Mbp) was identified as QTL for albuminuria in a cross between B6 and D2 mice [38]. The genomic interval of *Tpir* overlaps with these QTL in Chr 2qF1-qH1, 129-158 Mbp (red bars).

Chr	Markers	cM	Chr	Markers	cM	Chr	Markers	cM
1	*D1Mit1002*	4.94	6	*D6Mit274*	23.7	13	*D13Mit60*	14.44
1	*D1Mit123*	17.63	6	*D6Mit3*	34.81	13	*D13Mit94*	24.5
1	*D1Mit213*	22.88	6	*D6Mit194*	62.9	13	*D13Mit66*	34.54
1	*D1Mit282*	37.02	6	*D6Mit201*	77.7	13	*D13Mit68*	41.38
1	*D1Mit191*	52.66	7	*D7Mit178*	2.02	13	*D13Mit262*	63.93
1	*D1Mit14*	67.71	7	*D7Mit117*	17.26	14	*D14Mit49*	7.08
1	*D1Mit355*	80.36	7	*D7Mit82*	32.76	14	*D14Mit121*	25.36
1	*D1Mit291*	88.97	7	*Tyr* (coat color)	49.01	14	*D14Mit225*	39.46
1	*D1Mit511*	97.3	7	*D7Mit222*	59.13	14	*D14Mit266*	64.86
2	*D2Mit1*	2.23	7	*D7Mit105*	70.29	15	*D15Mit175*	3.96
2	*D2Mit293*	17.24	8	*D8Mit124*	7.59	15	*D15Mit152*	17.41
2	*D2Mit369*	24.51	8	*D8Mit4*	18.89	15	*D15Mit270*	27.16
2	*D2Mit91*	39.24	8	*D8Mit234*	39.33	15	*D15Mit71*	37.8
2	*D2Mit66*	49.45	8	*D8Mit211*	52	15	*D15Mit108*	47.79
2	*D2Mit164*	60.69	9	*D9Mit2*	20.76	15	*Tns2*	57.29
2	*D2Mit282*	73.59	9	*D9Mit49*	34.32	16	*D16Mit182*	2.57
2	*D2Mit229*	88.99	9	*D9Mit113*	46.4	15	*D16Mit166*	21.4
3	*D3Mit164*	2.01	9	*D9Mit355*	51.41	16	*D16Mit59*	26.86
3	*D3Mit203*	10.82	10	*D10Mit298*	2.78	16	*D16Mit76*	38.95
3	*D3Mit182*	21.73	10	*D10Mit124*	9.75	16	*D16Mit152*	48.23
3	*D3Mit78*	48.81	10	*D10Mit196*	32.28	16	*D16Mit106*	57.68
3	*D3Mit291*	63.05	10	*D10Mit42*	39.72	17	*D17Mit135*	15.8
3	*D3Mit129*	80.49	10	*D10Mit264*	45.66-46.68	17	*D17Mit119*	38.15
4	*D4Mit235*	3.57	10	*D10Mit233*	61.58	17	*D17Mit187*	50.17
4	*D4Mit172*	16.7	10	*D10Mit271*	72.31	17	*D17Mit221*	59.77
4	*D4Mit139*	29.65	11	*D11Mit226*	5.64	18	*D18Mit132*	11.92
4	*D4Mit27*	42.13	11	*D11Mit230*	16.15	18	*D18Mit53*	28.28
4	*D4Mit31*	50.04	11	*D11Mit140*	32.13	18	*D18Mit186*	45.63
4	*D4Mit12*	57.76	11	*D11Mit4*	41.87	18	*D18Mit4*	57.33
4	*D4Mit13*	75.67	11	*D11Mit179*	54.6	19	*D19Mit60*	13.9
5	*D5Mit346*	2.62	11	*D11Mit199*	65.48	19	*D19Mit39*	23.68
5	*D5Mit352*	18.4	11	*D11Mit333*	71.83	19	*D19Mit19*	34.08
5	*D5Mit254*	30.56	11	*D11Mit48*	82.96	19	*D19Mit33*	51.76
5	*D5Mit201*	39.55	12	*D12Mit209*	6.2	X	*DXMit123*	3.19
5	*D5Mit338*	53.23	12	*D12Mit243*	15.71	X	*DXMit73*	33.5
5	*D5Mit370*	65.23	12	*D12Mit36*	26.43	X	*DXMit38*	57.4
5	*D5Mit122*	89.28	12	*D12Mit214*	37.86	X	*DXMit186*	76.75
6	*D6Mit86*	1.81	13	*D13Mit205*	3.08			
6	*D6Mit159*	12.36	13	*D13Mit17*	7.73			

Table S1. Genotyping markers for consomic analysis. B6 (aBC at these three loci for coat color) and ICGN (aBc). *Tyr*^{c} (MGI:1855976) is located on Chr 7 (49 cM). Coat color (black or albino) was used for the genotyping of this locus.

Chr	Markers	Mb	cM
2	*D2Mit1*	3.8	2.23
2	*D2Mit293*	25.2	17.24
2	*D2Mit64*	31.2	21.81
2	*D2Mit369*	40.6	24.51
2	*D2Mit241*	45.1	27.37
2	*rs33151033*	49.5	
2	*rs49812762*	50.6	
2	*D2Mit91*	66.5	39.24
2	*D2Mit378*	68.6	39.53
2	*D2Mit328*	71.9	42.89
2	*D2Mit271*	74.6	44.13
2	*D2Mit219*	75.4	44.69
2	*D2Mit185*	105.3	55.23
2	*D2Mit102*	114.1	57.65
2	*D2Mit62*	117.9	59.34
2	*D2Mit451*	155.4	77.26
2	*D2Mit409*	158.5	78.72
2	*D2Mit229*	168.6	88.99

Table S2. Genotyping markers for subcongenic strains.
